# Timing of palliative care referral and aggressive cancer care toward the end-of-life in pancreatic cancer: a retrospective, single-center observational study

**DOI:** 10.1186/s12904-019-0399-4

**Published:** 2019-01-28

**Authors:** Natasha Michael, Greta Beale, Clare O’Callaghan, Adelaide Melia, William DeSilva, Daniel Costa, David Kissane, Jeremy Shapiro, Richard Hiscock

**Affiliations:** 1 Palliative and Supportive Care Research Department, Cabrini Institute, 154 Wattletree Road, Malvern, VIC 3144 Australia; 2 School of Medicine, University of Notre Dame, Sydney, NSW Australia; 3 Faculty of Medicine, Nursing and Health Sciences, Monash University, Clayton, VIC Australia; 4 Departments of Psychosocial Cancer Care and Medicine, St. Vincent’s Hospital, The University of Melbourne, Melbourne, VIC Australia; 5 Institute for Ethics and Society, University of Notre Dame, Sydney, NSW Australia; 6 Pain Management Research Institute, Royal North Shore Hospital, Sydney, Australia; 7 Sydney Medical School, University of Sydney, Sydney, Australia; 8 Szalmuk Family Psycho-Oncology Research Unit, Cabrini Health, Melbourne, VIC Australia; 9 Mercy Hospital for Women, Melbourne, VIC Australia

**Keywords:** Pancreatic cancer, End-of-life, Palliative care, Place of death, Aggressive cancer care

## Abstract

**Background:**

Pancreatic cancer is noted for its late presentation at diagnosis, limited prognosis and physical and psychosocial symptom burden. This study examined associations between timing of palliative care referral (PCR) and aggressive cancer care received by pancreatic cancer patients in the last 30 days of life through a single health service.

**Method:**

A retrospective cohort analysis of end-of-life (EOL) care outcomes of patients with pancreatic cancer who died between 2012 and 2016. Key indicators of aggressive cancer care in the last 30 days of life used were: ≥1 emergency department (ED) presentations, acute inpatient/intensive care unit (ICU) admission, and chemotherapy use. We examined time from PCR to death and place of death. Early and late PCR were defined as > 90 and ≤ 90 days before death respectively.

**Results:**

Out of the 278 eligible deaths, 187 (67.3%) were categorized as receiving a late PCR and 91 (32.7%) an early PCR. The median time between referral and death was 48 days. Compared to those receiving early PCR, those with late PCR had: 18.1% (95% CI 6.8–29.4%) more ED presentations; 12.5% (95% CI 1.7–24.8%) more acute hospital admissions; with no differences in ICU admissions. Pain and complications of cancer accounted for the majority of overall ED presentations. Of the 166 patients who received chemotherapy within 30 days of death, 23 (24.5%) had a late PCR and 12 (16.7%) an early PCR, with no association of PCR status either unadjusted or adjusted for age or gender. The majority of patients (55.8%) died at the inpatient palliative care unit.

**Conclusion:**

Our findings reaffirm the benefits of early PCR for pancreatic cancer patients to avoid inappropriate care toward the EOL. We suggest that in modern cancer care, there can sometimes be a need to reconsider the use of the term ‘aggressive cancer care’ at the EOL when the care is appropriately based on an individual patient’s presenting physical and psychosocial needs. Pancreatic cancer patients warrant early PCR but the debate must thus continue as to how we best achieve and benchmark outcomes that are compatible with patient and family needs and healthcare priorities.

## Background

A diagnosis of pancreatic cancer is unsettling for patients and their families, with late presentations at diagnosis and new therapeutic agents offering only modest improvements in survival [1, 2]. The median overall survival of metastatic pancreatic cancer is 8–11 months and the median overall survival of locally advanced (but not metastatic) inoperable pancreatic cancer is 12–14 months [3, 4]**.** Currently, less than 5–7% of Australians diagnosed with metastatic disease survive beyond five years [5]. Patients often experience significant physical symptom burden, treatment side effects, and psychosocial burden leading to depression and anxiety [6]. Despite pancreatic cancer being the fourth leading cause of cancer death in the United States of America [7] and Europe [8] and the fifth leading cause of cancer death in Australia [9], few studies have examined the impact of palliative care on the quality of end-of-life (EOL) care received in this patient cohort [10, 11].

Quality EOL indicators to evaluate the use of aggressive treatments toward the EOL for cancer patients are increasingly recommended and endorsed by peak bodies, including the National Quality Forum [12] and the American Society of Clinical Oncology (ASCO) Quality Oncology Practice Initiative [13]. Traditionally, aggressive cancer care received toward the EOL can be defined as any of the following: use of chemotherapy in the last 14 [14] or 30 days [11] of life, emergency department (ED) presentation, acute hospital/intensive care unit (ICU) admission within 30 days of death or death in ICU, and late referral to hospice/palliative care services (≤3 months from referral to death) [15, 16]. Studies have shown that cancer patients experience more aggressive treatments toward the EOL when they are younger, diagnosed with hematological cancers, have distant metastatic disease, poor prognostic tumors, and are managed by oncologists and in teaching hospitals [11, 17, 18].

EOL care however, is enhanced in cancer patients when palliative care is integrated early and provided for a longer duration, particularly after discontinuing chemotherapy [19]. Nonetheless, reports of chemotherapy use for patients with varied cancer diagnoses within the last month of life remains wide-ranging from less than 8 to 45.5% [11, 20, 21]. A retrospective cohort study of 366 cancer patients found that those referred early to palliative care benefited at the EOL through more hospice inpatient utilization (74% versus 47%, adjusted *p* < 0.001) [22] and fewer emergency room visits (39% vs. 68%, p < 0.001), hospitalizations (48% vs. 81%, *p* < 0.003) and hospital deaths (17% vs. 31%, *p* = 0.004) in the last 30 days of life. Furthermore, when EOL care planning discussions occurred, patients received less acute care within 30 days of death (OR: 0.67; *p* = 0.025) [15].

Data relating specifically to the use of aggressive treatments toward the EOL for pancreatic cancer patients remain limited. American surveillance data (comparing data from 1992 to 1994 and 2004–2006) has shown that despite an increase in hospice enrollment of pancreatic cancer patients, admissions to ICU and chemotherapy use in the last month of life increased significantly from 15.5 to 19.6% (*p* < 0.0001) and 8.1 to 16.4% (*p* < 0.0001) respectively [23]. A Swiss study of 231 pancreatic cancer patients similarly showed that 24% of patients received chemotherapy in the last 4 weeks of life, with the median survival from last chemotherapy to death being 7.5 weeks (95% CI 6.7–8.4) [24]. Conversely a retrospective population cohort study of 5381 Canadian patients with advanced pancreatic cancer found that PCR was associated with less chemotherapy treatment (OR 0.34, 95% CI 0.25–0.46), fewer ICU admissions (OR 0.12, 95% CI 0.08–0.18), reduced emergency department visits (OR 0.19, 95% CI 0.16–0.23), and fewer hospitalizations near death (OR 0.24, 95% CI 0.19–0.31) [10]. Similarly, a Taiwanese study showed that pancreatic cancer patients receiving inpatient palliative care compared to acute hospital care were more likely to receive opioids (84.4% vs. 56.5%, respectively; *p* < 0.001), had shorter acute hospital stays (10.6 ± 11.1 days vs. 20.6 ± 16.3 days, respectively; p < 0.001), fewer aggressive procedures, and lower medical costs (both, *p* < 0.005) [25].

The poor survival outcomes and high symptom burden experienced in pancreatic cancer makes it the ideal prototype cancer to study the quality of EOL care. This study, conducted at a single health service, aimed to examine associations between timing of PCR and aggressive cancer care received by pancreatic cancer patients in the last 30 days of life.

## Methods

### Design

We conducted a retrospective observational study of a prevalent cohort of all patients registered with a diagnosis of pancreatic cancer between January 2012 and December 2016 in a single institution. We included patients over the age of 18 who were registered patients with Cabrini Health, who had received a referral to the palliative care service and who subsequently died. Follow up data was available until March 2017. Ethics approval was obtained from Cabrini Health’s Human Research Ethics Committee (Number: 06–19–06-17). The hospital’s administration provided consent to review patient records and utilize data for the purpose of this study. The Strobe statement was used as a guideline in preparation of this manuscript [26].

### Setting

The data was obtained from a large not-for-profit, private health care service providing acute, sub-acute and community based care across six campuses in Melbourne, Australia. The Health Service has 31 accredited medical oncologists consulting across two sites, both providing chemotherapy services through day oncology clinics. In 2016, there were 3398 new cancer diagnoses registered, along with 24,551 same day and 9367 overnight episodes of care. Specialist palliative care is provide via a large integrated service incorporating a 22 bed inpatient unit, community service, supportive care clinics and consult services at no cost to the patient.

### Data sources and outcomes

The hospital’s administrative database was used to identify eligible patients using the International Classification of Diseases [27] Code C25.0 to C25.9 and who had a death registered on the database (C25.0-C25.9 describes the diagnosis ‘malignant neoplasm of pancreas’ in more detail). Confirmatory data on further deaths were obtained through the state department’s register of deaths. We captured basic demographic variables (age, sex, country of birth, date and place of death).

Clinical electronic and written case records and the hospital chemotherapy drug administration database were subsequently examined to identify key indicators of aggressive cancer care in the last 30 days of life which included: intravenous chemotherapy use, multiple emergency department presentations and acute hospital admission (defined as ≥1), or intensive care admission (≥ 1). We included chemotherapy administration in external hospitals if these data were available in the clinical records, as patients may have chosen to receive treatment elsewhere. We further determined if referral to the hospitals’ palliative care service had occurred, the interval between referral to palliative care and death, and the place of death. We choose to define early palliative care based on the duration of continuity of palliative care before death [15]. Thus early and late PCR were defined as more than 90 days and less than or equal to 90 days before death respectively.

## Statistical analysis

Summary measures of patients’ socio-demographic characteristics were presented as mean (SD), median [25th - 75th percentile] or number (%) according to type and distribution. Unadjusted associations between PCR and measures of aggressive cancer care and place of death used likelihood ratio chi-squared statistic based on a univariable logistic model, and the associations were also tested using a multivariable logistic model to adjust for age and gender. Strength of association is presented as either odds ratio (OR) or risk difference (RD) and the associated 95% confidence interval. There was no missing data except for four patient files which did not have information on chemotherapy usage. No imputations were made. The two-sided significance level was set at 0.05 and no adjustment was made for multiple comparisons. Statistical analysis was performed using Stata v 15 statistical software [28].

## Results

We identified 457 patients with a diagnosis of pancreatic cancer over the study period. Of these, 278 met the eligibility criteria of being registered with the health service with a diagnosis of pancreatic cancer, receiving a referral to the hospital’s palliative care service, more than 18 years of age and dying within the study period. Patient characteristics by PCR status are presented in Table 1. Compared to patients receiving late PCR those with an early PCR were younger, with mean difference 5.1 (95%CI 2.2 to 8.0) years. The median time between referral and death was 48 days, with 187/278 (67.3%) of patients categorized as receiving a late PCR and 91/278 (32.7%) receiving an early PCR.

**Table 1:** Patient characteristics by palliative care referral

	Palliative Care Referral (Days before death)		
Characteristic	Early (> 90 days)	Late (≤ 90 days)	*p*-value
	(*n* = 91)	(*n* = 187)	
Age (years)	73 (11.6)	78 (11.7)	< 0.001
Gender (male)	40 (44.0%)	95 (50.8%)	0.28
Marital Status			
Married	59 (64.8%)	110 (58.8%)	0.11
Widowed	18 (19.8%)	47 (25.1%)	
Single	7 (7.7%)	11 (5.9%)	
Divorced / Separated	7 (7.7%)	10 (5.4%)	
Defacto	0	7 (3.7%)	
Unknown	0	2 (1.1%)	
Place of Birth			
Australia / New Zealand	58 (63.7%)	122 (65.3%)	0.83
United Kingdom / Ireland	9 (9.9%)	15 (8.0%)	
Other European Countries	14 (15.4%)	32 (17.1%)	
Asia	2 (2.2%)	6 (3.2%)	
Others	8 (8.8%)	11 (5.9%)	
Unknown	0	1 (0.5%)	
Time between Palliative Care referral and death			
Median [25th - 75th percentile] days	177 [133–295]	26 [11–49]	
Place of Death			
Palliative Care Unit	50 (18%)	105 (37.8%)	0.98
Acute Hospital	22 (7.9%)	44 (15.8%)	
Home/Residential Care Facility	19 (6.8%)	38 (13.7%)	

Data presented as mean (SD), median [25th – 75th percentile] or count %

Emergency department presentation, acute hospital and intensive care unit admission.

Measures of aggressiveness of cancer care are summarized in Table 2. One hundred and one (36.3%) patients presented to the ED within the last 30 days of life and, of these, 15 (14.9%) had more than one admission. Those with a late PCR were 18.1% (95%CI 6.8–29.4%, *p* = 0.003) more likely to have an ED presentation than those with an early PCR. Reasons for ED presentation were widely varied (see Table 2) with main reasons including pain, nausea, vomiting and complications of cancer (e.g. biliary obstruction and ascites) and infection. One hundred and seventy (61.2%) patients had an admission to the acute hospital in the last 30 days of life, with 19 (6.8%) having more than one admission. There is moderate evidence that late PCR is associated with a 12.5% (95%CI 1.7–24.8%, *p* = 0.04) higher acute hospital admission rate compared to early PCR (Table 3). Furthermore, the number of admission episodes is increased in those receiving late PCR compared to those receiving early PCR in the last 30 days of life (Table 2). Based upon a total of 191 admission episodes, 31.4% were discharged home, 38.7% transferred to an inpatient palliative care unit and 28.8% died in hospital. Only 3 (1.1%) patients had an ICU admission in the last 30 days of life and there were no deaths in the ICU. There was also no association between the timing of PCR and patients’ place of death (Table 3).

**Table 2:** Aggressiveness of care in the last 30 days of life for 278 patients with pancreatic cancer

	Palliative Care Referral (Days Before Death)		
	Early (> 90 days)	Late (≤ 90 days)	*p*-value
	*N* = 91	*N* = 187	
Emergency Department (ED) Presentations			
No of ED presentations			
Total number of patients	22 (24.2%)	79 (42.2%)	0.003
No of patients presenting once	19 (86.3%)	67 (84.8%)	0.57^a^
No of patients presenting twice	2 (9.1%)	11 (13.9%)	
No of patients presenting thrice	1 (4.6%)	1 (1.3%)	
Total number of presentations	26	92	
Reason for ED presentation (based upon all presentations in last 30 days of life)^b^			
Pain	9 (34.6%)	19 (20.7%)	
Nausea/Vomiting	1 (3.8%)	16 (17.4%)	
Confusion	1 (3.8%)	8 (8.7%)	
Other Symptoms	7 (26.9%)	8 (8.7%)	
Infection	3 (11.5%)	8 (8.7%)	
Complications of Cancer	6 (23.1%)	22 (23.9%)	
Investigations and Procedures	0	1 (1.1%)	
Funtional decline	0	4 (4.3%)	
Other medical problems	0	9 (9.8%)	
End of life care	1 (3.8%)	0	
Outcome of ED presentation			
Admit to acute hospital	18 (81.8%)	66 (83.6%)	0.26
Discharge home from ED	1 (4.6%)	8 (10.1%)	
Transfer to palliative care unit	0	2 (2.5%)	
Death in ED	3 (13.6%)	3 (3.8%)	
Acute Hospital Admissions by Patient			
No of acute hospital admission			
Total number of patients	48 (52.8%)	122 (65.2%)	0.04
0	43 (47.3%)	65 (34.7%)	0.009
1	47 (51.6%)	104 (55.6%)	
2	1 (1.1%)	16 (8.6%)	
3	0	2 (1.1%)	
Total admission episodes	49	142	
Outcome of acute hospital admission (total admission episodes)			
Discharge home from ward	12 (24.5%)	48 (33.8%)	0.31
Transfer to palliative care unit	19 (38.8%)	55 (38.7%)	
Transfer to another hospital	0	2 (1.4%)	
Death on ward	18 (36.7%)	37 (26.1%)	
Intensive Care Admission	1 (1.1%)	2 (1.1%)	0.98
Chemotherapy Use			
Received any chemotherapy^c^	73 (80.2%)	97 (51.9%)	< 0.001
Received Chemotherapy < 30 days^d^	12/72 (16.7%)	23/94 (24.5%)	0.22

Data presented as number (%)

^a^Testing hypothesis of a difference in number of presentations, in patients who present to ED.

^b^Patients may present with ≥1 reason to ED

^c^Figures relate to any chemotherapy, not just < 30 days

^d^Excludes 4 patients with unknown chemotherapy status

**Table 3:** Odds ratios (OR) for aggressive management comparing late to early (referent level) Palliative Care referral

	OR (95%CI, p- value)	
	Unadjusted	Adjusted for age & gender
Emergency Department presentation	2.29 (1.31 to 4.02, *p* = 0.004)	1.92 (1.08 to 3.42, *p* = 0.03)
Acute Hospital Admission	1.68 (1.01 to 2.80, *p* = 0.05)	1.60 (0.95 to 2.69 *p* = 0.08)
Intensive Care Admission	0.97 (0.09 to 10.90, *p* = 0.98)	1.09 (0.09 to 12.74, *p* = 0.94)
Received any Chemotherapy	0.27 (0.15 to 0.48, *p* < 0.001)	0.33 (0.17 to 0.66, *p* < 0.002)
Received Chemotherapy < 30 days before death	1.6 (95%CI 0.74 to 3.53, *p* = 0.22)	1.7 (95%CI 0.75 to 3.7, *p* = 0.21)
Place of death		
Palliative Care Unit (referent level)	1	1
Home / RCAF	1.00 (0.47 to 2.12, *p* = 0.99)	1.06 (0.48 to 2.32, *p* = 0.88)
Acute Hospital	1.05 (0.57 to 1.94, *p* = 0.88)	1.11 (0.59 to 2.09, *p* = 0.76)

### Chemotherapy use

**Fig. 1:**
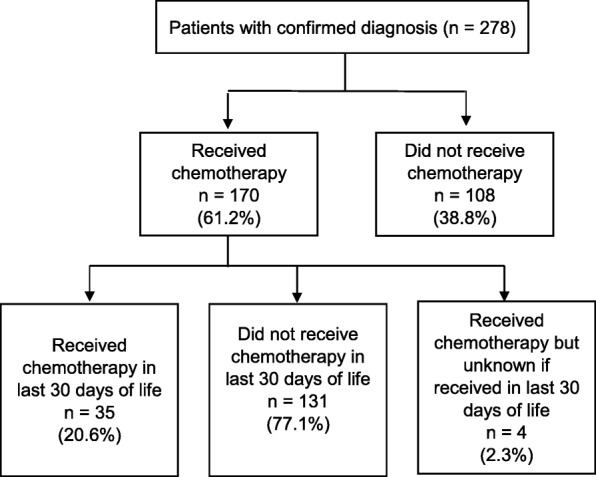
Chemotherapy use in the last 30 days of life

Details of chemotherapy use are summarized in Fig. 1. Overall 170 (61.2%) patients were recorded as having received intravenous chemotherapy at any one time during the study period. Those patients with early PCR were more likely to receive chemotherapy at any time compared to those with late PCR [risk difference 28.3% (95%CI 17.4 to 39.2, *p* < 0.001)] (Table 3). Overall, 108 (38.8%) did not receive chemotherapy due to advanced nature of the illness at diagnosis, high risk due to comorbidities or patient refusal. Details for the use of chemotherapy within 30 days of death was available for 166 patients. Twenty-three patients (24.5%) received chemotherapy with late PCR and 12 (16.7%) with early PCR within 30 days of death. There was no association of chemotherapy usage within 30 days of death and PCR status either unadjusted or adjusted for age or gender (Table 3).

## Discussion

This is the first Australian study to examine EOL outcomes in a cohort of pancreatic cancer patients referred to a palliative care service and its association with timing of PCR. Given that a recent systematic review demonstrated a 98% loss of healthy life in pancreatic cancer patients [29], it is paramount that due vigilance is given to the EOL care experience of patients and caregivers. The ASCO practice guidelines for metastatic pancreatic cancer recognizes palliative care as an important adjunct in management and recommends early initiation of a referral, preferably at the first visit [6]. This recommendation is further validated by findings from a recent multicenter Delphi study undertaken to establish an international core set of patient reported outcomes (PROs) in pancreatic cancer [30]. Eight PRO’S rated as ‘very important’ by patients (curative- and palliative-setting) and health care professionals were: general quality of life, general health, physical ability, ability to work/do usual activities, fear of reoccurrence, satisfaction with services/care organizations, abdominal complaints (pain/discomfort) and relationship with partner/family.

There remains ambiguity as to what constitutes an early referral to palliative care, with figures ranging from > 3 months before death [15, 22, 31] to 6–14 months prior to death [32]. Only a third of our patient cohort (32.7%) received an early PCR (> 3 months before death). This compares to 10.1% of 922 [31] and 33% of 366 [15] patients with all cancer types in single American institutions. Additionally our median time between referral to death was 48 days. This compared to Bennett et al. who found the median duration of palliative care involvement before death across 3 services in the United Kingdom was 37 days, with differences in duration identified between cancer and non-cancer patients (16 versus 22 days) and setting of care (community or the acute hospital; 22 days versus 13 days). There was a statistical difference (p = < 0.001) between cancer types, with prostate or breast cancer having the longest time (median days of 43.5 and 48 days respectively) and hematological and head and neck cancers having the shortest time (median 26 days) [32]. A similar retrospective Irish study conducted across an integrated palliative care service found that mean time from referral to death interval was 70 days, with the majority receiving care across more than one setting [33].

Studies have also demonstrated that longer referral-to death interval increases likelihood of dying at home or in an inpatient hospice [34] and the intensity of palliative care follow up is associated with fewer instances of aggressive treatments used near death, with the minimum number of palliative care contacts needed to benefit ranging between three and four [10]. Additionally, home has been shown to be the preferred place of death for the majority of cancer patients [35] and those never admitted to an inpatient palliative care unit, whilst those with at least one admission to an inpatient palliative care unit have shown preference for care in this setting [32]. Despite our findings demonstrating no association between timing of PCR and place of death, the majority (55.8%) of patients died in the inpatient palliative care unit, with only a fifth (20.5%) dying at home/residential care facility. We did not collect data on the intensity of palliative care follow up following referral that may have influenced this outcome. In this cohort with pancreatic cancer, the location for death for a pancreatic cancer patient may have been influenced by poorly controlled symptoms and complications of cancer as detailed in Table 2, necessitating the availability of expert care. The additional perceived burden on others and security with the familiarity of staff and environment [36] may have been contributing factors. Our high death rates in the inpatient palliative care unit may also be attributed to our model of early integration palliative care [37], which facilitates admission for symptom control and rehabilitation through the illness trajectory and not simply EOL care. 38.7% of those admitted into hospital were subsequently transferred to the inpatient palliative care unit, thus increasing the likelihood of patients having one or more admissions to an inpatient palliative care unit pre-death, possibly influencing preference for care in the this setting toward the EOL.

When evaluating quality indicators for care of cancer patients in their last days of life, Raijmakers et al. found that > 80% of respondents agreed that < 4% of patients who die should have > 1 ED visit, > 1 hospitalizations or have an ICU admission in the last 30 days [38]. Our findings were contrary to these recommendations, with just over a third (36.3%) of patients presenting to the ED and close to two thirds (61.2%) having an acute hospital admission in the last 30 days of life. Additionally, 14.9% had > 1 ED presentations and 6.8% > 1 hospital admission. Nonetheless, ED presentations and hospital admissions remain common in patients with advanced cancer. A systematic review and meta-analysis of 30 studies by Henson et al. examined ED attendance by cancer patients in their last month of life [39]. It described contributing demographic, clinical and environmental variables that increased the likelihood of ED presentations which included being male and of black race, having lung cancer, lower socioeconomic status and without a PCR [39]. Worsening symptoms, treatment toxicity and caregiver stress have also been shown to contribute to ED visits [40] as found in our study (Table 2). Nonetheless, our findings supported the findings of Hui et al. [15], confirming benefits of early palliative care, with a late PCR increasing the likelihood of ED presentations and number of hospital admissions in this cohort.

**Fig. 2:**
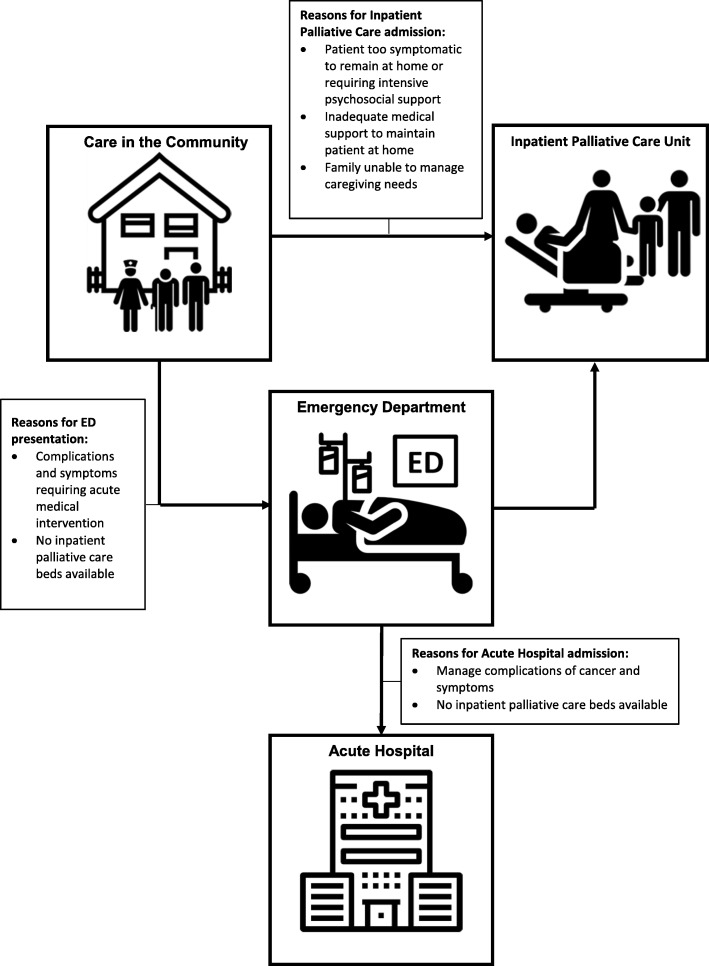
Patient flow through an integrated model of care Images depicted in Fig. 2 were adapted from icons by Freepik, Smartline and Smashicons from www.flaticon.com

These contradictory findings of seemingly aggressive cancer care supports the findings of Wijnhoven et al. who in a qualitative study exploring the impact of incurable cancer (pancreatic and non-small cell lung cancer) on family members and primary care-givers suggests that ED presentations and hospital admission may be considered to be part of necessary treatment as opposed to avoidable burdens [41]. Figure 2 demonstrates the integrated model of care at the study site, demonstrating how symptom complexity, psychosocial distress, lack of inpatient palliative care beds or staffing may lead to ED or acute hospital presentations. Patient and caregiver strain can be significant when patients experience the array of symptoms and complications that may arise with pancreatic cancer as shown in our findings. Pain is associated with reduced survival in pancreatic cancer and affects up to 80% of patients, with 50% requiring strong opioid analgesia [42]. Depression together with anxiety affects 33–50% of patients [43], influencing overall quality of life and the social, emotional and functional wellbeing of both patient and caregiver. Complications may include obstruction, ascites and thromboembolism [6], all of which cause significant symptomology and require acute medical intervention.

Finally, the use of chemotherapy in situations that are deemed futile remains common in cancer, with its use sometimes being justified as a means to reinforce hope in dire situations [44]. This collusion of hope may be unnecessary if honest conversations and early involvement of palliative care service are used to assist patients and families come to terms with the inevitable outcome. In our study, we failed to categorize the cohort to those with potentially curable, locally advanced or metastatic disease and failed to exclude those with a diagnosis of an additional cancer. These factors may have contributed to chemotherapy use in the small cohort of 35 (20.6%) of the 170 patients who received treatment in the last 30 days of life.

## Conclusion

Our findings mirror the results of a small number of international studies and reaffirm the benefits of early referral to palliative care for pancreatic cancer patients to avoid futile treatment and inappropriate care toward the EOL [10, 23, 25]. We however question the current benchmarks for aggressive cancer care at the EOL, based on our findings that patients with significant symptoms and whose caregivers lack support appropriately require acute hospital service utilization or care in a supported environment. We suggest that in modern cancer care, there can sometimes be a need to reconsider the use of the term ‘aggressive cancer care’ at the EOL when the care is appropriately based on an individual patient’s presenting physical and psychosocial need [45]. For pancreatic cancer patients, the wide spectrum of significant symptomology experienced and the condensed time frame associated with the diagnosis may appropriately justify the use of acute services and treatments at this point of life.

Ironically, the widespread use of what is traditionally described as aggressive treatments in the final month of life may paradoxically rise with palliative care integration earlier in the disease trajectory and into the acute setting, as symptom burden is appropriately managed, unless outreach community services develop alternate solutions to reduce hospital presentations and maintain care in the community. The debate must thus continue as to how we best achieve and benchmark outcomes that are compatible with patient and family needs, informed views, experiences and healthcare priorities.

## Abbreviations

ASCO: American Society of Clinical Oncology
CI: Confidence Interval
ED: Emergency Department
EOL: End-of-life
ICU: Intensive Care Unit
OR: Odds Ratio
PCR: Palliative Care Referral
PRO: Patient Reported Outcomes
RD: Risk Difference
SD: Standard Deviation
